# The Effect of CaCl_2_ on the Gelling Properties of Pea Protein–Pectin Dispersions

**DOI:** 10.3390/gels11010018

**Published:** 2024-12-31

**Authors:** Dan Zhang, Da Chen, Osvaldo H. Campanella

**Affiliations:** 1 Department of Food Science and Technology, The Ohio State University, 2015 Fyffe Rd., Columbus, OH 43210, USA; zhang.11473@osu.edu; 2 Department of Food Science, Purdue University, 745 Agriculture Mall Drive, West Lafayette, IN 47907, USA; chen3370@purdue.edu

**Keywords:** plant protein gels, CaCl_2_, rheology, microstructure, interactions

## Abstract

The effects of CaCl_2_ addition before (PreCa) or after (PostCa) heating pea protein–pectin dispersions on the formed gel’s rheological and microstructural properties were investigated. Isothermal titration calorimetry (ITC) revealed that CaCl_2_ bound both pea proteins and pectins through a spontaneous exothermic reaction and pectin exhibited a stronger binding affinity to CaCl_2_. In PreCa gels, low levels of CaCl_2_ (5 and 10 mM) increased the gel elasticity (increase in the storage modulus, G′) and their microstructural compactness. However, higher CaCl_2_ levels (15 and 25 mM) decreased gels’ elasticity, likely due to diminished hydrogen bonds formed in the cooling stage, resulting in gels with larger voids and fewer interconnections between the protein and pectin phases. In PostCa gels, their elasticity increased with the CaCl_2_ content, a rheological change associated with the formation of denser microstructures. The addition of 25 mM CaCl_2_ decreased *β*-sheet and increased *α*-helix and random coil structures. Hydrogen bonding and electrostatic and hydrophobic interactions contributed to gel formation and stability in both PreCa and PostCa gels, whereas disulfide bonds had negligible effects. This study highlights the role of CaCl_2_ in modulating pea protein–pectin gels’ properties and microstructures for the development of gel-like foods with diverse textures and mouthfeels.

## 1. Introduction

Pea protein-based foods are arising in the market due to their non-genetic characteristics, hypoallergenic nature, and health benefits like antioxidant, antihypertensive, and intestinal bacteria-modulating activities [1,2]. Gels are a predominant type of protein-based food characterized by a high-water content and appealing texture and mouthfeel, and are ideal carriers for bioactive compounds [3,4]. Heating is a commonly used method to induce protein gelation on a large scale. Heat-induced protein gelation primarily involves the partial unfolding and exposure of the protein’s non-polar and sulfhydryl groups, followed by aggregation and agglomeration via covalent and non-covalent interactions, such as electrostatic interactions, van der Waals forces, hydrogen bonds, and hydrophobic interactions [5].

Like most plant proteins, pea proteins exhibit a relatively weak gel strength compared to their animal counterparts, primarily due to their poor water solubility and low cystine content [2]. To address these limitations, incorporating polysaccharides, such as pectin, cellulose, *κ*-carrageenan, gellan, and curdlan gum, has been an effective strategy [6,7,8,9]. For instance, the addition of 0.5% pectin increased the storage modulus of pea protein gels by nearly six times [10]. Beyond improving gel properties, pectin is recognized as a safe additive and dietary fiber that can provide nutritional benefits [11]. The dual functional and nutritional roles of pectin make it a promising additive for improving pea protein gels.

The textural and microstructural properties of pea protein–pectin (PP-Pec) gels can be influenced by various factors, such as the use of other biopolymers and the ratio used, pH, and the presence of ions. The effects of biopolymer ratios and the pH were investigated in our previous studies [10,12], but the effect of ions is still unclear. The presence of ions mainly affects the electrostatic interactions between charged groups. Specifically, divalent ions like Ca^2+^ are known to be highly effective in forming ionic crosslinks, and their presence would introduce complexity to the molecular interactions within the PP-Pec systems, as both the protein and pectin can independently interact with Ca^2+^ or with each other [13]. As a result, the gel’s physical and microstructural characteristics, which are key for food applications, can be altered by the presence of Ca^2+^ [3].

The effect of CaCl_2_ content on gel properties has already been studied in many protein–polysaccharide composite gels, such as *Mesona chinensis* polysaccharide–whey protein isolate [3], *Mesona blumes* polysaccharide–soy protein isolate [14], wheat starch–whey protein isolate [5], and whey protein isolate–lotus root amylopectin [15]. However, whether there is any difference based on the order of CaCl_2_ addition, for instance, before or after the heating of protein–polysaccharide dispersions, remains largely unexplored. Adding CaCl_2_ before the heating process might change the protein status and its denaturation behavior when present in a dispersion. The denaturation behavior would alter the extent of protein unfolding and chain orientation in the heating process, which would ultimately change the properties of the final gel products [13]. Conversely, adding CaCl_2_ after heating mainly affects the molecular interactions formed during the gelation step and the gelation rate, both of which are believed to affect the physical properties of protein–polysaccharide gels.

Therefore, the objective of this study was to investigate the effects of CaCl_2_ concentrations and the timing of addition (before or after the heating process) on the gelling properties of PP-Pec mixed gels. Understanding the effect of Ca^2+^ on the gel properties is essential for optimizing the formulation and processing conditions of gel products with diverse quality attributes.

Regarding the calcium incorporation, CaCl_2_ concentrations ranging from 5 mM to 25 mM were selected in this study based on their nutritional relevance. Calcium is an essential dietary mineral, and its supplementation in food products can help alleviate calcium deficiency [13]. This range closely aligns with typical calcium concentrations reported in food products, which range from 10 mM to 30 mM [16]. To ensure a moderate calcium intake, the maximum CaCl_2_ concentration was set at 25 mM. At this concentration, a serving size of 250 g of PP-Pec mixed gels would contain approximately 200 mg of calcium, providing a valuable dietary calcium source. This level helps consumers to meet calcium needs while staying within the recommended daily intake for adults (1000 mg, according to the Dietary Reference Intakes) when consumed alongside other calcium-rich foods such as milk, cheese, and yogurt [17].

Isothermal titration calorimetry was for the first time used to investigate CaCl_2_–pea protein and CaCl_2_–pectin interactions. The viscoelasticity and microstructures of gel-forming dispersions were analyzed using a rheometer and confocal microscopy. After gelation, the rheological properties, molecular forces, and microstructures of gels, as well as secondary structure changes in pea proteins, were investigated.

## 2. Results and Discussion

### 2.1. The Effect of CaCl_2_ on the Physical Properties of PP-Pec Dispersions

Considering that the physicochemical properties of biopolymer gels are directly related to their gel-forming dispersion properties prior to gelation, the viscosity and microstructures of gel-forming dispersions used to prepare PreCa gels were first investigated. The characteristics of the gel-forming dispersions used to prepare PostCa gels were unchanged before the heating process, so they were not subjected to these analyses.

**Figure 1:**
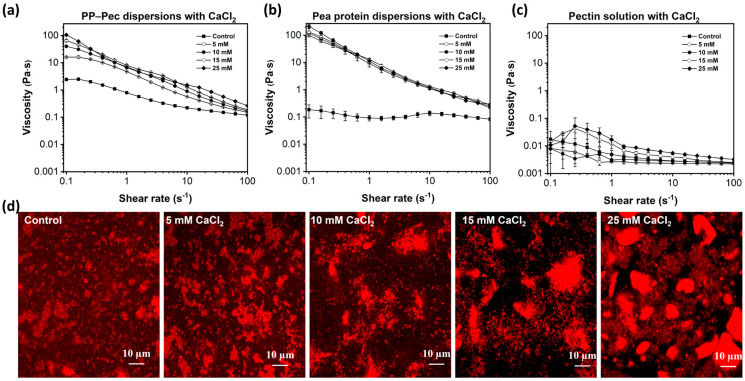
Viscosity of pea protein–pectin dispersions (**a**), pea protein dispersions (**b**), and pectin solutions (**c**) in the presence of different concentrations of CaCl_2_, along with the confocal microscopy of pea protein–pectin dispersions with various CaCl_2_ contents (**d**).

The influences of different CaCl_2_ concentrations on the viscosity of PP-Pec dispersions, pea protein dispersions, and pectin solutions are shown in Figure 1a, Figure 1b, and Figure 1c, respectively. At all concentrations of CaCl_2_ tested, the viscosity of PP-Pec dispersions decreased with an increasing shear rate, indicating their shear-thinning behavior. The addition of CaCl_2_ increased the viscosity of dispersions in a concentration-dependent way at a low shear rate (<0.3 s^−1^). At a high shear rate, the viscosity of PP-Pec dispersions initially increased with the CaCl_2_ concentration but decreased at the highest content (25 mM). CaCl_2_ had a limited effect on the viscosity of the pectin solution (Figure 1c), whereas around a 500 times fold increase in viscosity was found in pea protein dispersions after adding 5 mM of CaCl_2_ (Figure 1b). Thus, the increased viscosity of PP-Pec dispersions after adding CaCl_2_ might be mainly ascribed to the increased protein–protein interactions caused by the presence of Ca^2+^, which promoted the aggregation of the proteins, and an increase in the size of the protein aggregates was observed through confocal microscopy (Figure 1d).

Ca^2+^-induced protein aggregation was thought to arise from three effects, including promoting hydrophobic effects in the proteins, the crosslinking of adjacent anionic groups via a protein–Ca^2+^–protein bridge, and electrostatic shielding [18]. Specifically, CaCl_2_ could compete with pea proteins to absorb water for hydration, thus changing the protein water environment, inducing protein unfolding and leading to protein aggregation through hydrophobic interactions. The presence of Ca^2+^ might also induce crosslinking between proteins by bridging negatively charged groups or neutralizing some of the charges on the protein surface, which decrease electrostatic repulsion and increase protein–protein interactions [3,14]. In addition, it is worth noting that the presence of calcium ions might decrease the pH of the dispersions by the ions binding to carboxyl groups and releasing hydrogen ions [19]. The pH of the 10% *w*/*w* pea protein water-soluble extract solutions was reported to decrease from 7.1 to 6.4 upon the addition of 20 mM CaCl_2_ [19]. The reduced pH after CaCl_2_ addition might serve as another driving force for the formation of larger pea protein aggregates in the PP-Pec dispersions, since the repulsive forces among the pea proteins decreased. Moreover, it is worthy to note that the viscosity of the PP-CaCl_2_ system was higher than that of the PP-Pec-CaCl_2_ system for the same protein and CaCl_2_ contents, which suggests that the presence of pectin decreases the increase in viscosity observed in the pea protein system. This phenomenon could be attributed to the competitive binding of pea proteins and pectins to Ca^2+^. Pectin could bind with Ca^2+^, leaving less available Ca^2+^ to induce pea proteins’ aggregation, resulting in the lower viscosity observed in the PP-Pec-CaCl_2_ dispersion when compared to the viscosity of the PP-CaCl_2_ dispersion. Similar results were reported for a whey protein–pectin system [20].

The pea protein water solubility in PP-Pec dispersions was also measured. The results showed that the addition of 5, 10, 15, and 25 mM CaCl_2_ decreased the pea protein solubility from 10.4 ± 0.4% to 9.1 ± 0.6%, 8.5 ± 0.1%, 7.6 ± 0.5%, and 6.6 ± 0.3%, respectively. The decreased solubility can mainly be ascribed to the calcium ion-induced formation of larger and insoluble aggregates observed using confocal microscopy.

### 2.2. CaCl_2_–Pea Protein and CaCl_2_–Pectin Interactions

**Figure 2:**
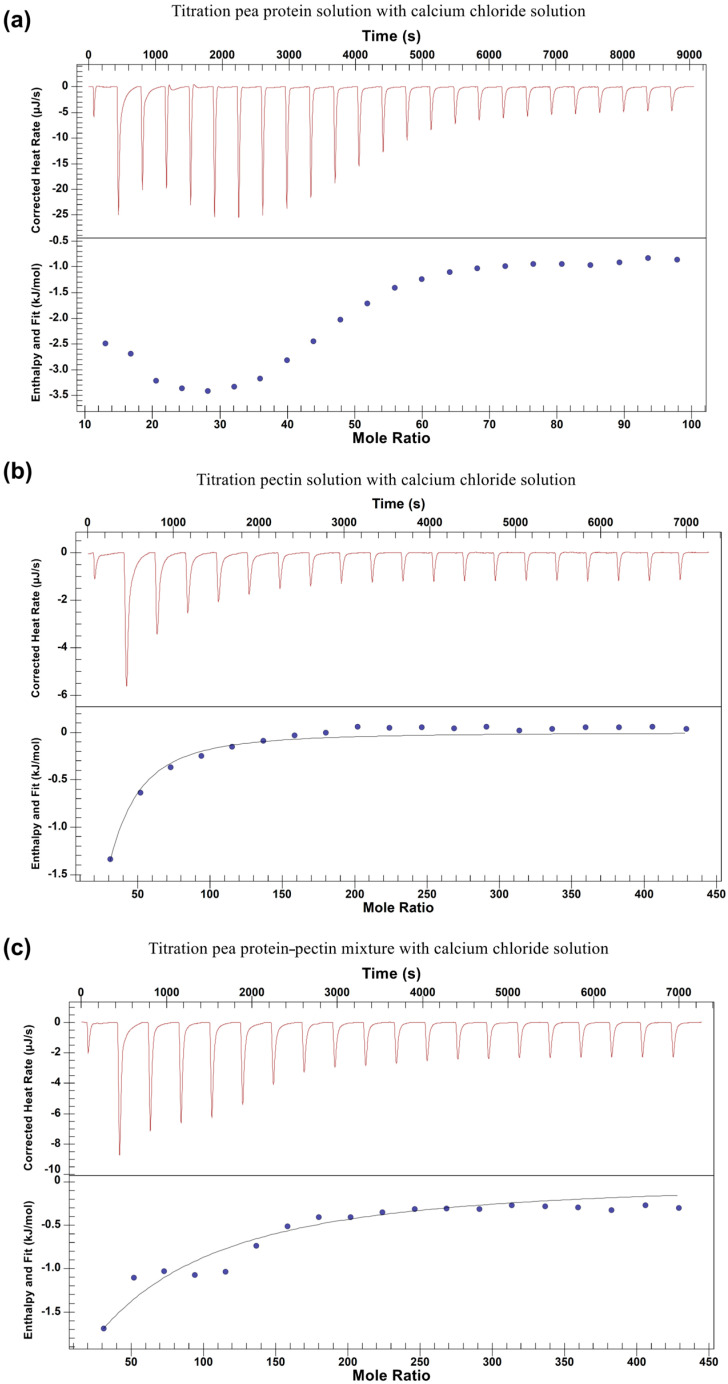
Isothermal titration calorimetry data for CaCl_2_ titration into pea protein solutions (**a**), pectin solutions (**b**), and pea protein–pectin mixtures (**c**) at pH 7 and 25 °C. The upper panels show the heat measured per injection as a function of the time after baseline correction, and the bottom panels show the integrated enthalpy change as a function of the molar ratio of CaCl_2_ to pea protein (**a**), CaCl_2_ to pectin (**b**), and CaCl_2_ to the pea protein–pectin mixture (**c**). The solutions of pea protein, pectin, the pea protein–pectin mixture, and CaCl_2_ were prepared in a 50 mM, pH 7 Tris buffer, and their concentrations were determined to be 35.4 μM, 6.7 μM, 6.7 μM, and 20 mM, respectively.

ITC was used to further investigate the binding behavior in CaCl_2_–pea protein and CaCl_2_–pectin systems. ITC directly measures the energy associated with the molecular interaction at a constant temperature, allowing for the determination of the interaction-related thermodynamic parameters. The ITC raw data after baseline correction (upper panels) are shown in Figure 2. The area under each peak represents the heat exchange within the ITC cell after each injection. The corresponding bottom panels in Figure 2 show the integrated enthalpy change as a function of the molar ratio of CaCl_2_ to pea proteins or pectins.

In the ITC raw data, the heat measurements of the first two datapoints could be discarded since the system was unstable due to the diffusive mixing in the syringe tip during the baseline acquisition [21]. Generally, with the titration of ligands into macromolecule solutions, the accessible binding sites on the adsorbing macromolecule are gradually decreased, leading to progressively decreased absolute values of binding enthalpy until a state of thermodynamic saturation is reached [22]. However, as shown in Figure 2a, the CaCl_2_–pea protein system exhibited two-step exothermic binding behavior. During the first phase, the height of the exothermic peaks continuously increased with the injection of CaCl_2_ until a critical molar ratio of CaCl_2_–pea protein (around 25) was achieved, beyond which continuous titration decreased the height of the exothermic peaks until saturation was reached. The increased magnitude of exothermic peaks observed in Figure 2a might be ascribed to the pea protein’s conformational change to a more stable state. Similar two-step binding isotherms were observed in Bowman–Birk protease inhibitor–carrageenan [22], BSA–dextran sulfate [21], and pea globulin–egg white protein systems [23]. To calculate the thermodynamic parameters involved in the titration process, different binding models in the NanoAnalyze software (version 3.12.5) were used to fit the ITC curve. However, none of the ITC models used were suitable, mainly because the pea protein solution was a complex mixture of various protein fractions instead of purified proteins, which are more often studied. The complexity of the system investigated in the current work made the binding behavior and ITC curve interpretation more complicated. Moreover, the calorimeter measures all processes occurring simultaneously and the total heat of ITC measurements includes contributions from several phenomena, such as classic interactions, protein conformational changes, aggregations, etc. [23,24].

For the CaCl_2_–pectin titration system, as shown in Figure 2b, the binding between pectin and CaCl_2_ was exothermic, and the magnitude of the exothermic peaks became gradually smaller until complete saturation was reached due to the decreased availability of the pectin carboxyl groups. For this case, the ITC curve was fitted by an independent binding model, which assumed that all binding sites on pectins had the same binding affinity and all binding events were independent. The thermodynamic parameters, including the binding affinity stoichiometry (*n*), binding affinity constant (*Ka*), Gibbs free energy change (Δ*G*), enthalpy change (Δ*H*), and entropy change (Δ*S*), were calculated to be 10 mol Ca^2+^/mol pectin, (7.54 ± 0.99) × 10^3^ M^−1^, −22.1 ± 0.13 kJ/mol, −9.84 ± 0.29 kJ/mol, and 41.2 ± 1.18 J/mol, respectively. The results indicated that 1 mol of pectin would bind 10 mol of Ca^2+^, with a binding event occurring spontaneously (Δ*G* < 0). Additionally, the binding between CaCl_2_ and pectin was controlled by both enthalpy- and entropy-driven processes (Δ*H* < 0, Δ*S* > 0). Binding enthalpy mainly reflects the binding strength between the ligand (CaCl_2_) and the target macromolecule (pectin). The low-methxyl pectin (DE of 6.7%) used in this study comprised consecutive blocks of non-methyl-esterified galacturonic acid (GalA) residues, and at pH 7, the carboxylate groups on GalA became ionized and negatively charged, promoting strong electrostatic interactions with Ca^2+^ [25]. Furthermore, the increased disorder (Δ*S* > 0) observed in the titration process could be ascribed to the water release after the complex formation between Ca^2+^ and the carboxylate groups, resulting in a disrupted water molecule structure surrounding the pectin chains [25].

The molar concentration of the PP-Pec mixture used in the ITC test was 6.7 μM, and the mixture contained equal molar amounts of pea proteins and pectins. As shown in Figure 2c, for the PP-Pec-CaCl_2_ titration, the overall exothermic binding enthalpy gradually decreased until saturation, with the curve showing a concave shape up to a molar ratio of approximately 150, which is similar to the curve shape observed for the first few injections in the CaCl_2_–pea protein titration. The curve shown in Figure 2c exhibits characteristics of both the CaCl_2_–pea protein and CaCl_2_–pectin titrations, indicating that CaCl_2_ could bind both pea proteins and pectins present in the PP-Pec mixture. This is consistent with the lower viscosity exhibited by the PP-Pec sample when compared with the only pea protein sample in the presence of the same amount of CaCl_2_ (Figure 1), which might be explained by a competitive binding of pea proteins and pectins to Ca^2+^.

An independent binding model was used to fit the ITC curve, and the thermodynamic parameters including *n*, *Ka*, Δ*G*, Δ*H*, and Δ*S* were calculated to be 5.37 ± 2.76 mol Ca^2+^/mol mixture, (1.38 ± 0.49) × 10^3^ M^−1^, −17.76 ± 0.86 kJ/mol, −75.52 ± 28.29 kJ/mol, and −193.68 ± 94.88 J/mol, respectively. The results indicated that the binding of CaCl_2_ with the PP-Pec mixtures occurred spontaneously (Δ*G* < 0) and the binding process was enthalpically favorable (Δ*H* < 0, Δ*S* < 0). The negative change in entropy could be ascribed to the decreased conformational freedom of the pea proteins and pectin after CaCl_2_ binding [23]. Moreover, related parameters such as the binding affinity stoichiometry (*n*) and the constant (*Ka*) were lower than those calculated for the CaCl_2_–pectin titration, suggesting that the presence of pea protein in the PP-Pec mixture reduced the numbers of molecules that CaCl_2_ could bind with. This may indicate that the presence of pectin provides more binding sites for CaCl_2_, resulting in a stronger measured binding strength than that measured between CaCl_2_ and a single pea protein. The zeta potential for pea protein and pectin at pH 7 was estimated to be around −12 mV and −30 mV, respectively, which demonstrated that pectin in solution has more negatively charged reactive sites [12]. Therefore, the greater number of negatively charged reactive sites of pectin might explain these results.

### 2.3. The Effect of CaCl_2_ on the Rheological Properties of PP-Pec Gels

**Figure 3:**
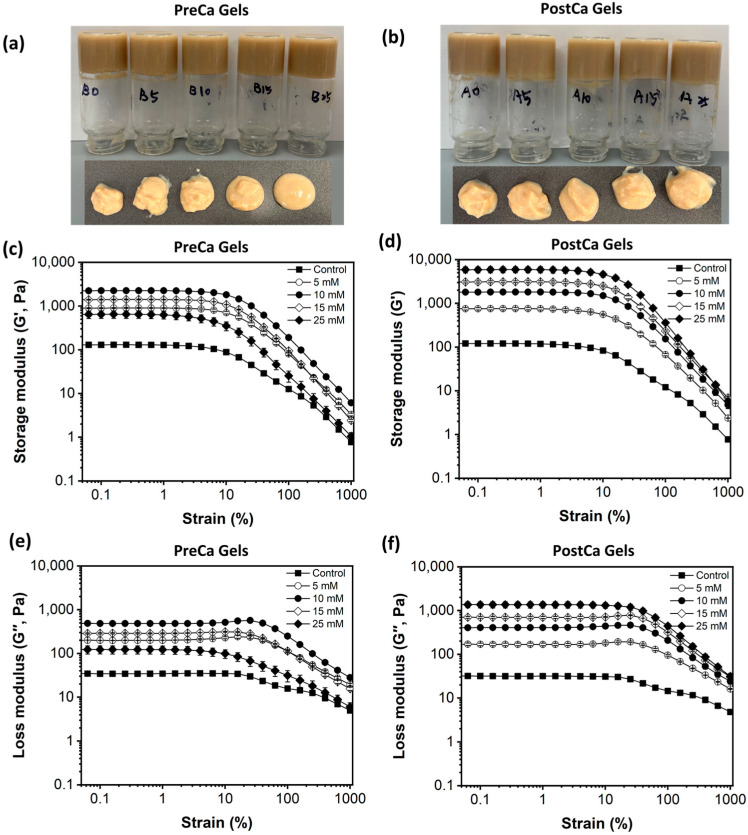
Appearance (**a**,**b**) and amplitude sweeps (**c**–**f**) of PreCa and PostCa gels.

PP-Pec gels including various concentrations of CaCl_2_ were formed after thermal-induced gelation, and the appearance of the gel samples is shown in Figure 3. All gels appeared to be opaque with a yellowish color. They were able to support themselves when the tubes were turned upside down. However, when external forces were applied, such as those applied when the samples were scooped out of the tubes, the gels lost their structural integrity and could not maintain a regular shape, instead behaving with a consistency like yogurt. There was no visible difference in the texture and consistency of the PostCa gels, which exhibited a relatively thick and solid consistency, while the PreCa gels containing 15 and 25 mM CaCl_2_ were less elastic and less firm.

The rheological properties of the samples were measured, and the results are shown in Figure 3. The results showed that the storage modulus (G′) was higher than the loss modulus (G′′) in all gel samples, suggesting that the gels exhibited an elastic nature. G′ often represents the formation and strength of a gel structure, and the increase in G′ indicates an improvement in the mechanical properties of the gel that is related to the strengthening of the gel structure. In the PreCa gels, the addition of low levels of CaCl_2_ (5 and 10 mM) increased the gels’ G′, while high levels (15 and 25 mM) decreased G′. A similar result was reported for *Mesona blumes* polysaccharide–soy protein gels, in which G′ values increased at low Ca^2+^ concentrations (5–10 mM) but decreased at higher concentrations (15–20 mM) [14].

The major difference observed in the PreCa gels was the size of aggregates found in their gel-forming dispersions (Figure 1b). These results suggest that the formation of gels with desirable rheological properties required an optimal aggregation extent or aggregate size that could be controlled by the amount of Ca^2+^. Typically, protein aggregates may interact strongly through protein–protein interactions that help to form thicker protein gel chains during the gelation process, thus enhancing the gel strength [26]. However, high amounts of Ca^2+^ could lead to the excessive aggregation of pea proteins, reducing the availability of interaction sites that are necessary for forming an interconnected gel network, disrupting the continuity of the gel matrix and weakening the overall gel strength. Moreover, the reduced water solubility of pea proteins in the presence of high concentrations of CaCl_2_ might also result in fewer proteins being available to participate in the formation of the gel network, ultimately leading to final gels with lower elasticity [26].

Figure 3d shows the effects of the CaCl_2_ concentration on G′ for PostCa gels. In these gels, the protein molecules first lost their native structures, with partial unfolding and subsequent aggregation during the heating process. The addition of CaCl_2_ could promote the interactions of heat-induced aggregates that shield charged groups and decrease their electrostatic repulsion. It could also act as a bridge to allow for crosslinks between negatively charged groups on pea proteins and pectins, leading to the formation of strong and well-interconnected gel structures. It should be noted that the preparation of PostCa gels is slightly different from a salt-induced cold gelation process, in which a stable mixture of protein aggregates is obtained by heating native globular proteins at a low ionic strength, followed by a gel network formation step performed by adding salts [27]. The protein concentration used for cold gelation should be below the critical gel concentration to avoid the formation of thermal gels during the initial heating process [28].

Furthermore, PostCa gels showed a similar (at 5 and 10 mM of CaCl_2_) or higher (at 15 and 25 mM of CaCl_2_) gel strength compared to PreCa gels in the presence of the same amount of CaCl_2_. Similar results were reported in heat-induced whey protein gels formed by adding 10 mM of CaCl_2_ or 200 mM NaCl before the heating process. The formed gels exhibited a lower gel strength compared to the gels formed by adding salts into pre-heated whey protein suspensions [29]. The different rheological properties found in PreCa and PostCa gels might be ascribed to the difference in the protein states (i.e., unfolded vs. native) prior to the salt addition, as well as the gelation speed [30]. The denatured protein might show higher binding sites and binding affinity to Ca^2+^ due to the exposure of previously buried functional groups. For instance, a native whey protein isolate was reported to be able to bind two Ca^2+^ ions per protein, while denatured whey protein could bind approximately three Ca^2+^ ions per protein in excess CaCl_2_ [27].

**Figure 4:**
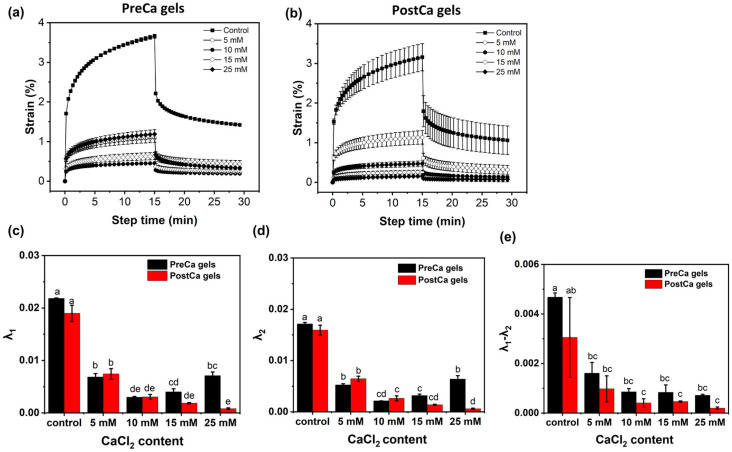
Creep–recovery curves (**a**,**b**) and fitted parameters (**c**–**e**) of PreCa and PostCa gels. Inverse of λ_1_ and λ_2_ are representative of the elastic moduli during creep and recovery, respectively; λ_1_–λ_2_ indicates the recovery capacity of the gel.

The effects of the CaCl_2_ contents on the creep–recovery behavior of both PreCa gels and PostCa gels were also assessed, and the results are shown in Figure 4. The PreCa gels without CaCl_2_ exhibited the highest values of creep and recovery strain, whereas moderate levels of CaCl_2_ (5 and 10 mM) decreased their creep strain, suggesting that the addition of a moderate amount of CaCl_2_ promoted the formation of stronger gels. However, higher concentrations (15 and 25 mM) had the opposite behavior, increasing their creep strain, indicating the formation of softer and more deformable gels. After fitting the data with the fractional calculus model, two parameters, including the inverse of the elastic modulus in creep (λ_1_) and recovery (λ_2_), were obtained. Lower values of λ_1_ and λ_2_ indicate higher gel elasticity [31]. Additionally, the difference (λ_1_–λ_2_) was calculated to evaluate the recovery capacity of the gels. The large observed difference indicates that gels cannot recover from deformation after the stress is removed, suggesting a poor recovery capacity of gels [32]. Gel samples without CaCl_2_ showed the highest values of λ_1_ and λ_2_, whereas moderate levels of CaCl_2_ (5 and 10 mM) decreased them and higher levels of CaCl_2_ (15 and 25 mM) increased them again. This suggests that the elasticity of the gels could be controlled with the concentrations of CaCl_2_. The effects of the CaCl_2_ content on the gels’ creep–recovery behavior was consistent with the amplitude sweep results. Additionally, the value of λ_1_ was always higher than λ_2_, indicating that the internal gel structures were disturbed during the creep test. As a result, gels could not recover to their original undeformed state once the stress was removed. The value of λ_1_–λ_2_ was reduced in the presence of CaCl_2_, indicating that Ca^2+^ might help to reduce structural disturbances caused by the application of stress. No significant difference in λ_1_–λ_2_ was observed in gel samples containing different concentrations of CaCl_2_. In PostCa gels, the addition of CaCl_2_ decreased the gels’ creep and recovery strain, along with decreasing the λ_1_, λ_2_, and λ_1_–λ_2_ values, indicating that the elasticity and recovery capacity of the gels were improved. This result was also consistent with the amplitude sweep results of PostCa gels.

Overall, the rheology and physicochemical results demonstrate that the timing of CaCl_2_ addition could affect the influence of the CaCl_2_ concentration on the gel strength and elasticity. The structures of protein gels largely depend on the rate of protein denaturation and the intermediate aggregation of denatured proteins prior to the formation of a gel network [33]. When CaCl_2_ was added to native state PP-Pec dispersions, its crosslinking effect altered the status of the protein’s conformation in the dispersions. This change would further affect the rate and extent of protein denaturation, leading to the formation of gels with different structures and physical properties depending on the balance between the denaturation and aggregation rates. Conversely, adding CaCl_2_ after heating allowed the proteins to denature and partially aggregate first, and then the addition of CaCl_2_ facilitated crosslinking to consistently enhance the gel strength with increasing concentrations of up to 25 mM.

### 2.4. Gelation Dynamics of PreCa Gels and PostCa Gels

Thus, it is important to gain a better understanding of the gel formation process and to identify at which point the gel strength of PreCa gels with a high CaCl_2_ content (15 and 25 mM) began to decrease. To address this challenge, a dynamic oscillatory shear test was conducted to monitor the changes in the viscoelastic behavior of gel-forming dispersions during the gelation process. This test is non-destructive and was conducted in the linear viscoelastic region (strain, 0.1%; frequency, 1 Hz).

**Figure 5:**
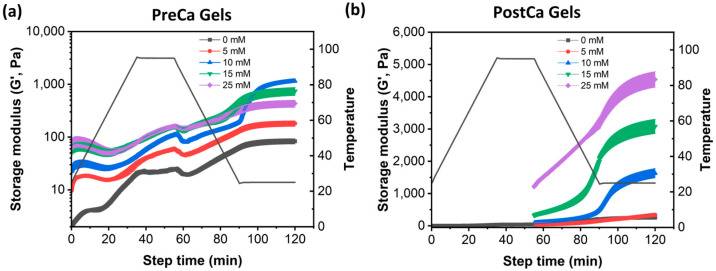
Temperature sweep of PreCa gels (**a**) and PostCa gels (**b**) at different concentrations of CaCl_2_.

The results are shown in Figure 5. In the PreCa gels, G′ increased slightly during the heating process and the incubation period at 95 °C. This was due to the unfolding of pea proteins and the exposure of groups that were initially buried within protein structures. These changes in the protein conformation tend to form aggregates through hydrophobic interactions, electrostatic interactions, and hydrogen bonding, leading to an increase in G′ [34]. In addition, a dramatic increase in G′ was observed during the cooling process and the incubation period at 25 °C, which was mainly attributed to the formation of hydrogen bonds and more ordered structures, further enhancing the strength and rigidity of protein networks [35]. It was observed that the addition of low CaCl_2_ concentrations (5 and 10 mM) increased the G′ of PP-Pec dispersions during the gelation process. The higher G′ could be attributed to CaCl_2_-induced protein aggregations, which increased the protein–protein and protein–pectin interactions. In addition, the results showed that in the presence of high concentrations of CaCl_2_ (15 and 25 mM), the increase in G′ slowed down at the late cooling stage, which might have been due to the decreased hydrogen bonds within the gel samples. High CaCl_2_ concentrations could induce the formation of excessive pea protein aggregations and raise their denaturation temperature. As a result, the degree of denaturation of pea proteins would be reduced, preventing the sufficient exposure of its reactive groups, thus reducing the formation of hydrogen bonds during the cooling stage, so that the gels would tend to show structures of large protein coagulates rather than a well-connected three-dimensional network [14].

Figure 5b demonstrates that the presence of CaCl_2_ led to a concentration-dependent increase in the G′ of the PostCa gels during the cooling stage. This phenomenon could be attributed to the crosslinking capacity of Ca^2+^. Pea proteins in dispersions were first denatured during the heating process, and the addition of CaCl_2_ facilitated the crosslinking between protein and protein molecules, as well as protein and pectin molecules, thereby enhancing the strength of the gel network. Moreover, the increased availability of Ca^2+^ at higher concentrations promoted more extensive crosslinking, leading to a stronger and more rigid gel structure.

### 2.5. Molecular Forces in PreCa Gels and PostCa Gels

**Figure A1:**
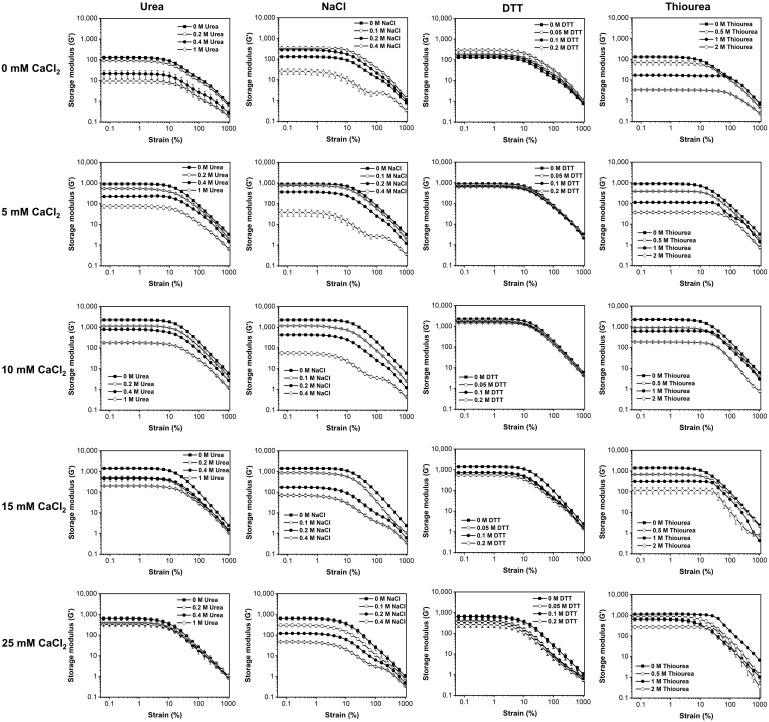
Amplitude sweeps of pea protein–pectin composite gels (CaCI_2_ added before heating) in the presence of various dissociation reagents, including NaCl, urea, thiourea, and DTT. The figures in the same column present gel samples treated with the same dissociation reagent, and the figures in the same row present gel samples containing the same amount of CaCl_2_.

**Figure A2:**
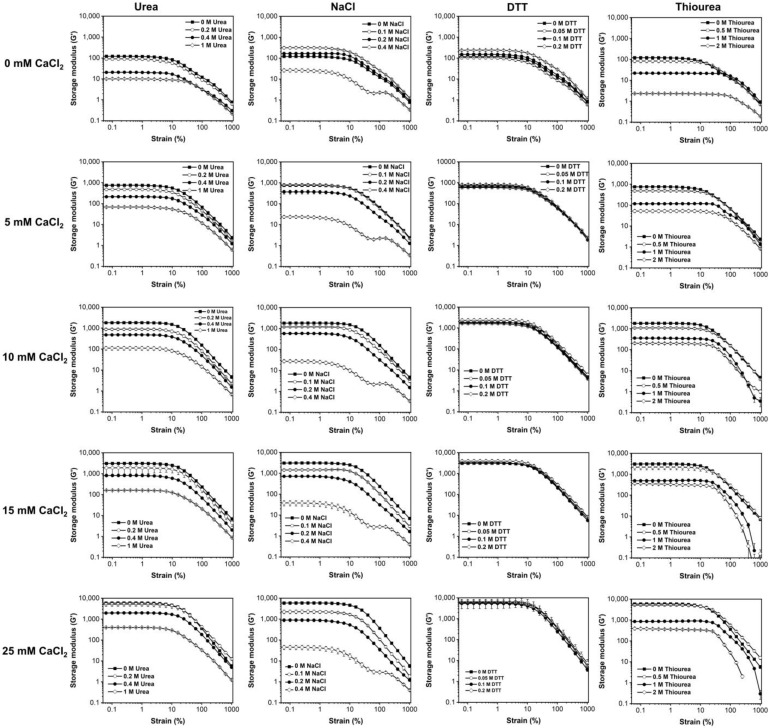
Amplitude sweeps of pea protein–pectin composite gels (CaCI_2_ added after heating) in the presence of various dissociation reagents, including NaCl, urea, thiourea, and DTT. The figures in the same column present gel samples treated with the same dissociation reagent, and the figures in the same row present gel samples containing the same amount of CaCl_2_.

To elucidate the molecular forces involved in the gel formation, various dissociation reagents were added to the PP-Pec dispersions before heat-induced gelation. These reagents and their molar concentrations were urea (0.2–1 M), NaCl (0.1–0.4 M), DTT (0.05–0.2 M), and thiourea (0.5–2 M). They can break down hydrogen bonds, electrostatic interactions, disulfide bonds, and electrostatic interactions within gel structures, respectively. An amplitude sweep test was employed to determine the G′ of the gels in the presence of dissociation reagents, and the results are shown in Figure A1 and Figure A2. The results indicated that, except with DTT, the G′ of gels decreased continuously as the concentration of the dissociation reagents increased due to the breakdown of molecular interactions. To present the results in a more concise and straightforward manner, only the effects of these reagents at their respective highest concentrations on the G′ of gel samples are reported in Table 1. The DTT results (Figure A1) revealed that the concentration had a relatively small effect on the G′ of gels compared with the other reagents, suggesting that gels were dominated by non-covalent interactions. Furthermore, in some cases, the addition of DTT (especially at higher concentrations) increased the G′ of gels. DTT is a powerful reducing agent, and small quantities of DTT can cleave intermolecular disulfide bonds, so a decrease in the strength and rigidity of gels is usually expected [33]. However, the destruction of disulfide bonds in native proteins could also promote the further unfolding and denaturation of pea proteins, and these unfolded proteins could interact more effectively due to the lack of restriction of movement imposed by the presence of disulfide bonds, thus leading to the formation of stronger gels [33], which would explain the increased G′ observed in our study. Similar results were reported by Havea et al. (2004), showing that whey protein gels containing DTT exhibited much higher G′ values (~29,000 Pa) than those of control whey protein gels (13,500 Pa) [33]. Hence, the effect of DTT on the G′ of gel samples at its lowest concentration (0.05 M) was selected and reported in Table 1.

The results of the PreCa gels showed that the addition of 1 M of urea reduced the G′ of PP-Pec gels containing 0–15 mM of CaCl_2_ by approximately 84.6–92.7%, whereas the G′ of PP-Pec gels containing 25 mM of CaCl_2_ only decreased by around 48%. These results show that hydrogen bonds were mainly involved in the gel formation of the PP-Pec dispersions containing low CaCl_2_ contents, but they were not major molecular forces responsible for maintaining the gel strength and stiffness in the gelled PP-Pec dispersions containing high CaCl_2_ contents. This result was consistent with the temperature sweep results, which showed that the increase efficiency in the gels’ G′ induced by CaCl_2_ in the cooling stage decreased significantly with high CaCl_2_ contents. This suggests that reduced hydrogen bonding might have been the main cause of the decreased gel strength and elasticity of PreCa gels with high contents of CaCl_2_ (15 and 25 mM). Additionally, the presence of 0.4 M NaCl and 2 M thiourea decreased the G′ of PreCa gels by 81.4–97.7% and 56.0–97.1%, respectively, whereas the addition of DTT resulted in a comparatively smaller reduction in G′, ranging from 15.6% to 60.6%, which indicates the involvement of electrostatic interactions and hydrophobic interactions in gel formation and a minor contribution from disulfide bonds. Similar results were also reported by Sun and Arntfield (2012), showing that non-covalent interactions including hydrogen bonds and electrostatic and hydrophobic interactions played key roles in the gel formation of pea proteins, while the role of disulfide bonds was nearly negligible [34]. These results can especially be explained by the low levels (~1%, based on dry protein) of cystine in pea proteins [36]. Furthermore, the presence of 0.4 M NaCl decreased the G′ of PreCa gels containing 0, 5, 10, 15, and 25 mM of CaCl_2_ by approximately 81.3%, 96.0%, 97.7%, 95.3%, and 93.1%, respectively, suggesting that the presence of CaCl_2_ increased electrostatic interactions participating in the formation of PreCa gels. Moreover, it should be noted that it was not practically possible to determine the relative contribution of each type of molecular force influencing the overall rheological properties of a specific gel based on these gel dissociation tests, because the concentration of each dissociation reagent and their ability to destroy the corresponding forces were different. However, the test served to identify the main interactions related to the gel’s rheological properties and ruled out others that were ineffective.

The results of PostCa gels show that the presence of urea, NaCl, and thiourea significantly decreased the G′ of gels from a range of 118.1–5893.3 Pa to 10.0–400.8 Pa, 23.9–41.9 Pa, and 2.3–359.0 Pa, respectively, suggesting that hydrogen bonding, electrostatic interactions, and hydrophobic interactions played key roles in influencing the gel strength and structure of PostCa gels. In addition, it is worth mentioning that the presence of 0.4 M NaCl decreased the G′ of gels containing 0, 5, 10, 15, and 25 mM of CaCl_2_ by approximately 79.8%, 97.1%, 98.7%, 98.9%, and 99.3%, respectively. This result indicates that increasing the CaCl_2_ content in PostCa gels increased electrostatic interactions or that electrostatic interactions played a more dominant role in maintaining the gel strength with high CaCl_2_ contents. Like with PreCa gels, the presence of DTT showed limited effects on the G′ values of PostCa gels, suggesting that the disulfide bonds were not largely involved in determining the gel structures and their associated rheological properties.

### 2.6. The Effect of CaCl_2_ on the Microstructures of PP-Pec Gels

**Figure 6:**
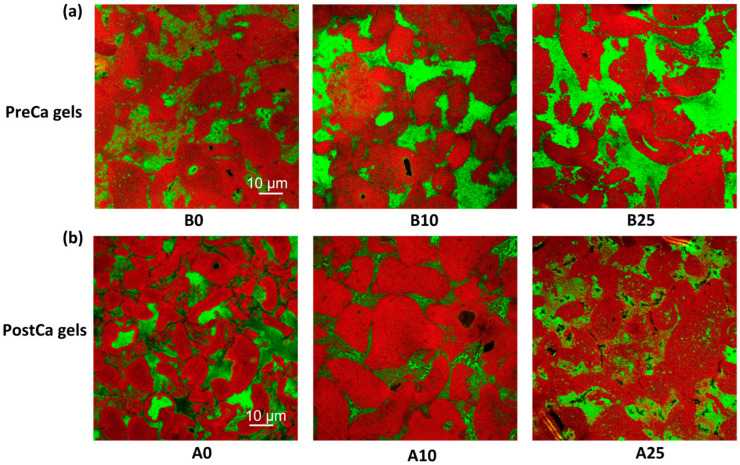
Confocal microscopy images of PreCa gels (**a**) and PostCa gels (**b**) containing various concentrations of CaCl_2_. “B” and “A” in the sample code refer to whether CaCl_2_ was added into the pea protein–pectin systems before (B) or after (A) the heating process. The numbers following the letters (0, 10, and 25) present the concentrations of CaCl_2_ in mM.

Confocal images of PreCa and PostCa gels are shown in Figure 6. Red areas indicate the pea protein phases stained by Nile blue. Pectin was labeled with antibodies (the monoclonal antibody LM19 and the secondary antibody goat anti-rat (H + L)) and appears in green.

The results show that PreCa gels without CaCl_2_ had a relatively uniform and interconnected network. In addition, the fluorescence of pea proteins and pectins exhibited some degree of phase separation and overlap, indicating both phase separation and interactions between the protein and the pectin. The figure also shows that a physical entrapment of pectin within a continuous pea protein network cannot be ruled out. As the concentration of CaCl_2_ increased, larger and more distinct protein aggregates appeared. This could be associated with enhanced interactions among pea proteins. Similar results were observed in *Mesona chinensis* polysaccharide–whey protein gels, showing that the presence of CaCl_2_ induced large agglomerates in the gel network [3]. Additionally, the voids in the protein network became larger and were primarily occupied by pectin. Consequently, the microstructure became more partitioned, and phase separation became more prevalent. The reduced interconnection among those large pea protein aggregates could help explain the low gel strength and elasticity of PreCa gels with high concentrations of CaCl_2_.

The results concerning the PostCa gel samples show that with increasing CaCl_2_ concentrations, the pea protein aggregates became larger, and a more compact and interconnected, less porous gel network was observed, which was believed to exhibit improved gel strength, and this was consistent with the rheology results. In addition, the fluorescence overlap area between pea proteins and pectins increased with an increasing concentration of CaCl_2_, which could be ascribed to the CaCl_2_-induced crosslinking of denatured proteins and pectins.

**Figure 7:**
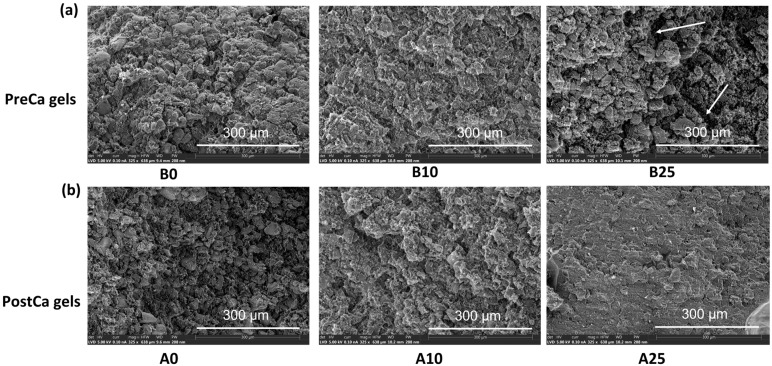
The SEM images of PreCa gels (**a**) and PostCa gels (**b**) containing various concentrations of CaCl_2_. “B” and “A” in the sample code refer to whether CaCl_2_ was added into the pea protein–pectin systems before (B) or after (A) the heating process. The numbers following the letters (0, 10, and 25) present the concentrations of CaCl_2_ in mM.

The microstructures of the gel samples were further investigated using SEM (Figure 7). SEM can provide more detailed and three-dimensional-like images of the sample surface morphology, which is not fully captured by confocal microscopy. It was observed that for PreCa gels, the addition of a low concentration of CaCl_2_ (10 mM) helped to form a more compact gel structure, while the structure became looser and the voids became larger with the addition of a high CaCl_2_ concentration (25 mM). Similar results were reported in whey protein isolate–lotus root amylopectin composite gels, showing that the addition of CaCl_2_ at concentrations ranging from 50 mM to 100 mM resulted in dense and homogeneous network structures, whereas increasing CaCl_2_ concentrations in a range from 150 mM to 200 mM led to larger cavities and irregular gel structures [15]. These results indicate that there is an optimal range of CaCl_2_ concentrations that allows one to optimize the structure and strength of protein–polysaccharide gels by keeping a balance between pea proteins and pectins and achieving a desirable extent of protein aggregation.

The results of PostCa gels showed that with an increasing concentration of CaCl_2_, the gel structures became highly compact and tight. The trends in the gel structure change with an increasing CaCl_2_ content observed using SEM were consistent with the observations using confocal microscopy.

### 2.7. The Effect of CaCl_2_ on the Secondary Structures of Proteins in PP-Pec Gels

**Figure 8:**
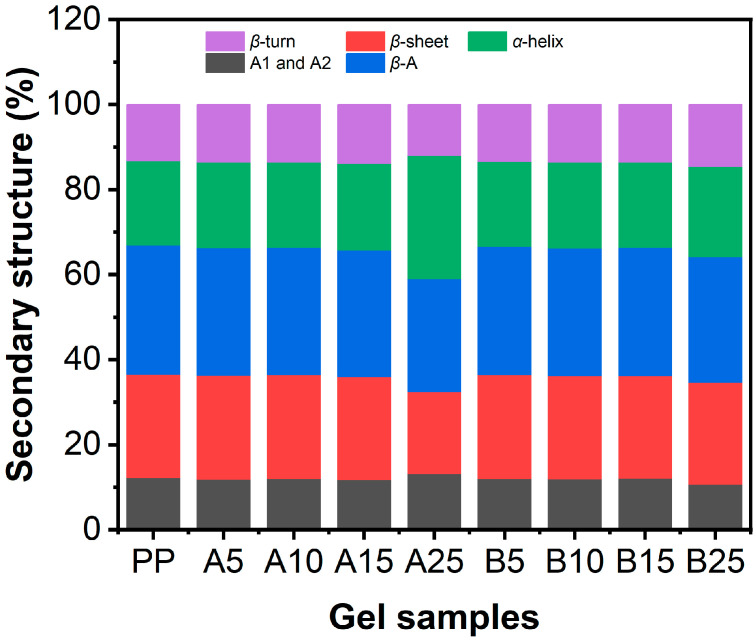
Secondary structure profiles of pea proteins in pea protein–pectin gels containing various concentrations of CaCl_2_. “B” and “A” in the sample code refer to whether CaCl_2_ was added into the pea protein–pectin systems before (B) or after (A) the heating process. The numbers following the letters (0, 5, 10, 15, and 25) present the concentrations of CaCl_2_ in mM.

The secondary structure profile of the gel samples was quantitively investigated. The amide Ⅰ region (1700–1600 cm^−1^) was deconvoluted and analyzed to estimate the secondary structural components of pea proteins since it is the most sensitive region to secondary structure changes [37]. Seven Gaussian peaks were observed and assigned to their corresponding structures based on their center. The peaks centered at approximately 1638 cm^−1^, 1645 cm^−1^, 1654 cm^−1^, and 1663 cm^−1^ correspond to the *β*-sheet, random coil, *α*-helix, and *β*-turn conformations, respectively [38,39]. Peaks in the range of 1610–1620 cm^−1^ (A1) and 1690–1695 cm^−1^ (A2) were assigned to protein aggregates as well as to the absorption of amino acid side chains [37,40]. Peaks centered at 1623 cm^−1^ and 1680 cm^−1^ were determined to be anti-parallel *β*-sheet (*β*-A) conformations [40]. These Gaussian peaks were assigned to their corresponding structures, and the relative proportions of different secondary structures were calculated by dividing the area under each Gaussian peak by the total area of all the peaks. The relative proportions of the secondary structures in all gel samples are shown in Figure 8. It was observed that, except the gel A25 (25 mM of CaCl_2_ was added after the heating process), all the other gels exhibited a similar secondary structure profile, with the largest contribution from *β*-structures, showing 24.0–24.3% of *β*-sheet, 29.6–30.4% of *β*-A, 13.2–14.6% of *β*-turn, 19.8–21.2% of *α*-helix and random coil, and 10.7–12.2% of A1 and A2 conformations. The predominance of *β*-structures in the secondary structure of pea protein was consistent with other studies that found that around 70% of the secondary structures of pea protein gels could be attributed to *β*-structures [37]. The gel sample A25, which as noted was prepared by adding 25 mM of CaCl_2_ after the heating process, showed a lower proportion of *β*-sheet (19.3%) and *β*-A structures (26.6%) and a higher content of *α*-helix and random coil (28.9%) and *β*-turn (12.0%) structures. Similar results were reported in different protein gel systems, including whey protein gel [41], peanut protein–basil seed gum gel [42], and mung bean protein–wheat gluten gel [43], showing that the incorporation of CaCl_2_ decreased the content of *β*-sheet structures and increased the content of random coil structures. Moreover, although more *β*-sheet conformations are generally considered favorable for forming larger and denser structures and increasing the gel strength, this phenomenon was not observed in our study. Instead, we found that the gels with the highest gel strength and elasticity exhibited relatively fewer *β*-sheet structures, suggesting that the positive relationship between *β*-sheets and gel strength might not be a universal rule.

## 3. Conclusions

This study found that the CaCl_2_ contents, as well as the order of CaCl_2_ addition and heating, could affect the rheological properties and microstructures of PP-Pec composite gels. In PP-Pec systems at pH 7, CaCl_2_ could bind both pea proteins and pectins, and the binding was controlled by a spontaneous exothermic process. Adding CaCl_2_ before heat-induced gelation could induce the formation of protein aggregates and increase the viscosity of the gel-forming dispersions in a concentration-dependent manner. The size of protein aggregates played an important role in determining the final gel properties, and moderate-sized aggregates induced by low CaCl_2_ contents (5 and 10 mM) could enhance the strength, elasticity, and compactness of PP-Pec gels due to increased protein–protein interactions. However, the formation of excess aggregates in gel-forming dispersions, induced by high CaCl_2_ contents (15 and 25 mM), might reduce the protein denaturation efficiency and the available soluble proteins for gelation, leading to the formation of weak gels with inhomogeneous and porous microstructures.

Adding CaCl_2_ after the heating process had no effect on the protein aggregates in the PP-Pec dispersions, but it could facilitate crosslinking between denatured proteins as well as between denatured proteins and pectins, resulting in the formation of strong and elastic gels with well-interconnected and dense microstructures.

A gel dissociation test revealed that hydrogen bonding, hydrophobic interactions, and electrostatic interactions were the predominant molecular forces maintaining the gel strength of PP-Pec gels, regardless of the order of CaCl_2_ addition, and the effect of disulfide bonds was negligible. The addition of CaCl_2_ both before and after the heating process increased the electrostatic interactions within the PP-Pec gels, while the addition of high CaCl_2_ concentrations (15 and 25 mM) before heating decreased hydrogen bindings. Moreover, the CaCl_2_ content and the timing of addition showed no significant effect on the secondary structures of pea proteins, except when adding 25 mM CaCl_2_ after the heating process, which resulted in a decrease in the content of *β*-structures and an increase in the *α*-helix and random coil content.

The findings of this research offer practical insights into the role of CaCl_2_ in modulating the rheological and microstructural properties of PP-Pec gels for different food applications. Low concentrations of CaCl_2_ (5–10 mM) added before heating, and a broader range of CaCl_2_ concentrations (5–25 mM) added after heating, are likely suitable for developing strong and elastic gels for applications such as plant-based yogurt, cheese, and encapsulation systems for bioactive compounds or probiotics. High concentrations of CaCl_2_ (15–25 mM) added before heating, on the other hand, are better suited for creating softer textures. These softer gels may find applications in plant-based spreads, puddings, low-calorie desserts, and protein-enriched sport gels, where a softer and less elastic texture is desirable. Future research could explore the application of CaCl_2_ in other biopolymeric systems to develop innovative and functional food products. In addition to improving the textural properties of gel-based foods, the CaCl_2_ levels selected would improve the nutritional qualities of these products.

## 4. Materials and Methods

### 4.1. Materials and Chemicals

Pea protein was purchased from ADM (Chicago, IL, USA), and the protein content was determined to be 76.8% using a nitrogen analyzer (Elementar, Germany); a conversion coefficient of 6.25 was used.

Low-methoxyl pectin (classic AU-L 057/21) was obtained from Herbstreigh & Fox. It was an unstandardized apple pectin with a molecular weight of 150 ± 17 KDa, a degree of esterification of 6.7%, and a galacturonic acid content of 72%.

CaCl_2_, potassium phosphate dibasic, potassium phosphate monobasic, and Rhodamine B were ordered from Sigma (St. Louis, MO, USA).

Hydrogen chloride, sodium hydroxide, urea, DTT, and thiourea were purchased from Fisher Scientific (Fair Lawn, NJ, USA). All chemicals were of analytical reagent grade unless otherwise noted.

### 4.2. Preparation of Gel-Forming Dispersions and Characterization

Pectin was weighted and dissolved in deionized water for 4 h with continuous stirring at room temperature and kept at refrigeration temperature overnight to ensure complete hydration. Pea protein was added to the pectin solutions and stirred at room temperature for 1 h. The pH of the mixed dispersions was adjusted to 7 using 2 N NaOH, followed by stirring for another 30 min. The final concentrations of pea protein and pectin were 18% (*w*/*v*) and 0.3% (*w*/*v*), respectively. Air bubbles were removed by centrifuging the PP-Pec dispersions at 138× *g* for 2 min. After that, 9.5 mL of the PP-Pec dispersions were transferred to 50 mL centrifuge tubes, and 0.5 mL of CaCl_2_ with various concentrations was added and gently mixed. The final concentrations of CaCl_2_ in PP-Pec dispersions were 5, 10, 15, and 25 mM. The same volume of deionized water was added to prepare a control sample.

The apparent viscosity of PP-Pec dispersions containing various CaCl_2_ contents was measured by using a Discovery HR-3 rheometer (TA instrument, New Castle, DE, USA) equipped with a 40 mm parallel plate geometry in a shear rate range of 0.1–100 s^−1^. Additionally, the viscosities of individual pectin solutions (0.3%, *w*/*v*) and pea protein dispersions (18%, *w*/*v*) were determined using the same method.

The soluble protein content in the PP-Pec dispersions was determined using the Bradford protein assay kit (ThermoFisher Scientific, Waltham, MA, USA). Specifically, PP-Pec dispersions were centrifuged (13,751× *g*, 10 min) to collect the supernatant, which was then diluted with deionized water. Subsequently, diluted samples (30 μL) were mixed with the Bradford reagent (1.5 mL) and allowed to react at room temperature for 10 min. After that, their absorbance at 595 nm was measured using a spectrophotometer (Fisher Scientific, Fair Lawn, NJ, USA). A calibration curve relating the measured absorbance to the protein content was constructed using bovine serum albumin (BSA) as the standard.

PP-Pec dispersions containing various CaCl_2_ contents were mixed well with Rhodamine B (0.2%, *w*/*v*). Then, 30 μL of these mixtures were loaded onto concave microscope slides and were sealed between the slide and cover slip using nail polish. The microstructures of the dispersions were observed using a 63× oil immersion objective on a Zeiss LSM900 confocal microscope (Oberkochen, Germany) at an excitation wavelength of 561 nm.

### 4.3. Isothermal Titration Calorimetry (ITC)

The heat flow resulting from the binding between CaCl_2_ and pea protein or pectin was examined using an isothermal titration calorimeter (MicroCal, Northampton, UK) according to the method reported by Wang, Munk, Skibsted, and Ahrné (2022) with small modifications [25]. In brief, pectin solutions (0.1%, *w*/*v*) were prepared in a 50 mM, pH 7 Tris buffer. Pea protein solutions (0.2%, *w*/*v*) were prepared by diluting the supernatants obtained from centrifuging the pea protein dispersions (18%, *w*/*v*) at 13,751× *g* for 30 min with a 50 mM, pH 7 Tris buffer. The molarity of pea protein and pectin solutions was determined to be around 35.4 and 6.7 μM, based on the molecular weight of pea protein (56 KDa) and pectin (150 KDa) (Zhang et al., 2022). In addition, mixtures of pea protein and pectin were prepared at a concentration of 6.7 μM by mixing equal volumes of pea protein solution (6.7 μM) and pectin solution (6.7 μM). The solutions of pea protein and pectin, as well as the mixture, were then filtered through 5 μm filters and dialyzed extensively in a pH 7, 50 mM Tris buffer for 24 h at room temperature. The dialysis buffer was used to prepare 20 mM CaCl_2_ solutions and was preserved for blank titrations. Prior to titration, all the solutions were degassed under vacuum for 30 min and the ITC200 instrument was stabilized at 25 °C. Subsequently, the CaCl_2_ solution loaded in a syringe was titrated into a cell containing either pectin solution (6.7 μM), pea protein solution (35.4 μM), or the PP-Pec mixture (6.7 μM). The titration was conducted with 25 successive injections (10 μL each injection) with an interval of 360 s between each injection. The stirring speed was set at 310 rpm to ensure full mixing during the test. Titrations were performed in triplicates at 25 °C. The blank titrations were conducted to obtain the generated dilution heat by injecting the CaCl_2_ solution into the dialysis buffer at 25 °C. The measured heat was then subtracted from the raw data to obtain the corrected enthalpy changes, so that the effects of sample mixing and dilution heat signals could be minimized. NanoAnalyze software (TA instrument, New Castle, DE, USA) was used to analyze the ITC data and an independent binding model was used to fit the data. Thermodynamic parameters including the binding affinity stoichiometry (*n*), binding affinity constant (*Ka*), Gibbs free energy change (Δ*G*), enthalpy change (Δ*H*), and entropy change (Δ*S*) were calculated.

### 4.4. Gel Preparation

To prepare PreCa gels, the PP-Pec dispersions containing various concentrations of CaCl_2_ were heated in a 95 °C water bath (Grant Instruments, Beaver Falls, AZ, USA) for 30 min, then cooled at room temperature and stored at 4 °C overnight. To prepare PostCa gels, 10 mL of PP-Pec dispersions were first heated at 95 °C for 30 min, followed instantly by adding 0.5 mL of the CaCl_2_ solutions and gentle mixing, and then they were cooled to room temperature and kept at 4 °C overnight. The final concentrations of CaCl_2_ in both PreCa gels and PostCa gels were set to be 5, 10, 15, and 25 mM. Gel samples were equilibrated at room temperature for 30 min prior to gel characterization.

### 4.5. Rheological Analysis

A Discovery Hybrid Rheometer 3 (DHR-3, TA Instruments, New Castle, DE, USA) equipped with a 40 mm parallel plate geometry was used to perform rheological experiments. Gel samples were loaded on the lower Peltier base, and the top plate was lowered down to a gap of 1 mm and rested for 2 min at 25 °C prior to the tests. The lineal viscoelastic range was determined by strain amplitude sweeps which were conducted at an oscillation frequency of 1 Hz using a strain range of 0.01% to 1000%; the storage (G′) and loss (G″) modulus were recorded during the test.

Creep–recovery tests were performed with an applied stress of 3 Pa for 30 min, followed by a 30 min recovery step. A water anti-evaporation accessory was used to minimize water loss. Data were fitted with a fractional calculus given by Equation (1) following Brito-Oliveira, Moraes, Pinho, and Campanella (2022) [31]:

(1) $$J\left(t\right)=\frac{\varepsilon \left(t\right)}{\sigma_{0}}=\frac{1}{\Gamma \;\left(\alpha +1\right)}\left(\lambda_{1}t^{\alpha }-\lambda_{2}{\left(t-t_{m}\right)}^{\alpha }H\left(t-t_{m}\right)\right)$$

*J*(*t*) represents the compliance of the material, measured in %/Pa. *ε*(*t*) is the instantaneous strain with the unit being the % strain. σ₀ is the stress applied during the creep phase, expressed in Pa. *t_m_* is the time in seconds taken to initiate the recovery test. The step function *H*(*t*) was employed to separate the creep curve into the creep and recovery regions and is defined as

(2) $$H\left(t\right)=\left\{\begin{array}{c} 1,\;t\geq 0\;\\ 0,\;t<0\; \end{array}\right.$$

The parameter *α* is an elasticity indicator. *λ*_1_ and *λ*_2_ represent the inverse of the gel elastic modulus during creep and recovery, respectively.

### 4.6. Gelation Dynamics—Temperature Sweep

The changes in the viscoelastic behavior of PP-Pec dispersions during gelation were evaluated using a Discovery Hybrid Rheometer 3 (DHR-3, TA Instruments, New Castle, DE, USA) equipped with a Peltier concentric cylinder geometry (cup diameter: 30.4 mm; bob diameter: 20 mm). The temperature sweep methods for the PreCa gels and PostCa gels were slightly different. For PreCa gels, 25 mL of PP-Pec dispersions containing various CaCl_2_ contents were loaded into the cup. The inner cylindrical bob was then lowered to a 5 mm gap between the base of the cup and the bottom of the bob. A water anti-evaporation accessory was used to minimize water loss. The dispersions were subjected to a temperature ramp from 25 °C to 95 °C at a heating rate of 2 °C/min, maintained at 95 °C for 20 min, then cooled to 25 °C at a cooling rate of 2 °C/min, and finally held at 25 °C for 30 min.

For PostCa gels, 25 mL of PP-Pec dispersions without CaCl_2_ were placed in the cup of the geometry and heated from 25 °C to 95 °C, then kept at 95 °C for 20 min. Subsequently, CaCl_2_ was added into the heated dispersions and gently mixed for several seconds. The mixtures were then cooled from 95 °C to 25 °C and held at 25 °C for 30 min. A water anti-evaporation accessory was used to minimize water loss. Throughout the temperature ramp treatment, the viscoelastic properties of the dispersions, specifically G′ and G″, were monitored using an oscillatory shear test conducted within a previously determined linear viscoelastic region (strain, 0.1%; frequency, 1 Hz).

### 4.7. Molecular Force Determination

Four different dissociation reagents, including urea, thiourea, DTT, and NaCl, were mixed well with PP-Pec dispersions. Their final concentrations were set in the ranges of 0.2–1 M, 0.5–2 M, 0.05–0.2 M, and 0.1–0.4 M, respectively. The reagents were then added into PP-Pec dispersions and gelled according to the method described in Section 2.4. Once the gel samples were ready, a strain amplitude sweep ranging from 0.01% to 1000% was conducted at 1 Hz to assess the lineal viscoelastic range and determine changes in the gels’ rheological properties in the presence of the reagents.

### 4.8. Confocal Laser Scanning Microscopy (CLSM)

CLSM was used to investigate the microstructure and distribution of pea proteins and pectins in the gels. Sections of the gel samples were prepared and stained according to the method described in a previous paper [10]. Images were observed using a Zeiss LSM900 microscope (ZEISS, Oberkochen, Germany). A 63× oil immersion objective lens was used with excitation wavelengths of 640 nm and 561 nm for the examination of protein and pectin, respectively.

### 4.9. Scanning Electron Microscopy (SEM)

Gel samples were prepared in 1 mL pipette tips and fixed with 2.5% (*w*/*w*) glutaraldehyde in 0.1 M of a phosphate buffer solution (PBS, pH 7.5) for 16 h. Subsequently, they were washed with PBS (2×, 30 min) and DI water (3×, 30 min). Afterwards, gel samples were dehydrated with a series of gradient ethanol–water solutions (25%, 50%, 70%, 85%, 95%, and 100%) and critical-point-dried. The dried gels were crushed to expose their fresh surface, then mounted on aluminum stubs and coated with 10 nm of a gold layer. The images of the gels were taken with a 325× magnification using a Quattro ESEM (ThermoFisher Scientific, Waltham, MA, USA) at an accelerating voltage of 6 kV and a low vacuum pressure of 40 Pa.

### 4.10. Fourier Transform Infrared (FTIR) Spectroscopy

The secondary structures of the gels were analyzed using an Agilent 4500 portable FTIR device equipped with a triple-reflection attenuated total reflectance (ATR) cell (Agilent Technologies, Santa Clara, CA, USA). Gel samples were directly replaced on the ATR crystal, followed by water evaporation using a faucet aspirator vacuum pump. The scan range, resolution, and scan number were set to 4000–600 cm^−1^, 2 cm^−1^, and 128, respectively. Three replicates were conducted for each sample. The spectra were subjected to Fourier self-deconvolution, second derivative analysis, and peak-fitting procedures to locate the overlapping peaks in the amide I region (1700–1600 cm^−1^) using PeakFit software (version 4.12, Systat Software, Palo Alto, CA, USA).

### 4.11. Statistical Analysis

All experiments were performed in three independent replicates. Statistical analysis was performed using OriginPro 2023b (Northampton, MA, USA). Multiple comparisons were carried out through a one-way analysis of variance (ANOVA) using the Tukey test with *p* < 0.05 as a significant level.

## Figures and Tables

**Table 1:** The storage modulus of pea protein–pectin gels in the presence of urea, NaCl, DTT, and thiourea, measured at a strain of 0.1%.

Sample Code	Storage Modulus (G′, Pa)				
	No Treatment	Urea (1 M)	NaCl (0.4 M)	DTT (0.05 M)	Thiourea (2 M)
B0	128.2 ± 8.7 ^a^	9.3 ± 2.3 ^c^	23.9 ± 7.7 ^c^	185.0 ± 8.4 ^b^	3.1 ± 0.1 ^c^
B5	900.3 ± 16.2 ^a^	74.7 ± 15.7 ^c^	35.3 ± 12.5 ^c^	637.0 ± 19.0 ^b^	37.3 ± 4.0 ^c^
B10	2241.2 ± 88.3 ^a^	177.1 ± 9.8 ^c^	51.7 ± 10.1 ^c^	1461.6 ± 93.5 ^b^	182.0 ± 2.2 ^c^
B15	1404.3 ± 21.0 ^a^	200.0 ± 9.8 ^c^	65.3 ± 5.0 ^d^	720.8 ± 51.7 ^b^	115.6 ± 38.9 ^cd^
B25	626.8 ± 122.8 ^a^	325.9 ± 78.2 ^b^	43.5 ± 4.7 ^c^	255.2 ± 67.3 ^bc^	275.9 ± 45.9 ^bc^
A0	118.1 ± 12.1 ^a^	10.0 ± 1.0 ^b^	23.9 ± 4.5 ^b^	101.9 ± 15.8 ^a^	2.3 ± 0.1 ^b^
A5	749.8 ± 44.9 ^a^	68.2 ± 5.1 ^c^	21.6 ± 2.9 ^c^	587.1 ± 82.9 ^b^	52.3 ± 9.4 ^c^
A10	1809.7 ± 157.0 ^a^	110.0 ± 24.4 ^b^	25.1 ± 4.3 ^b^	1614.1 ± 100.5 ^a^	197.6 ± 17.7 ^b^
A15	3103.7 ± 147.9 ^a^	159.9 ± 4.0 ^b^	35.0 ± 10.3 ^b^	3188.1 ± 336.6 ^a^	325.4 ± 36.1 ^b^
A25	5893.3 ± 927.8 ^a^	400.8 ± 10.5 ^b^	41.9 ± 10.7 ^b^	5835.4 ± 2755.1 ^a^	359.0 ± 20.2 ^b^

Note: “B” and “A” in the sample code refer to whether CaCl_2_ was added into the pea protein–pectin systems before (B) or after (A) the heating process. The numbers following the letters (0, 5, 10, 15, and 25) present the concentrations of CaCl_2_ in mM. Statistical analysis is performed within each row of the table. Values within the same row that are annotated with different superscript letters are significantly different (*p* < 0.05).

## Data Availability

The original contributions presented in this study are included in the article. Further inquiries can be directed to the corresponding authors.
